# Autistic-traits, not anxiety, modulate implicit emotional guidance of attention in neurotypical adults

**DOI:** 10.1038/s41598-019-54813-8

**Published:** 2019-12-05

**Authors:** Michael C. W. English, Murray T. Maybery, Troy A. W. Visser

**Affiliations:** University of Western Australia, School of Psychological Science, Perth, Australia

**Keywords:** Visual system, Human behaviour, Aggression

## Abstract

Although autistic and anxious traits are positively correlated, high levels of autistic traits are associated with poorer emotional guidance of attention (EGA) whilst high levels of anxious traits are associated with greater EGA. In order to better understand how these two trait dimensions influence EGA, we simultaneously examined the effects of anxiety and autistic traits in neurotypical adults on target identification in an attentional blink task. Analyses indicated that implicit EGA is attenuated in individuals with higher levels of autistic traits, but largely unaffected by variation in anxious traits. Our results suggest that anxiety plays a comparatively limited role in modulating implicit EGA and reinforces the importance of disentangling correlated individual differences when exploring the effects of personality, including emotional predisposition, on attention.

## Introduction

Resource limitations seriously affect our ability to simultaneously process sensory input. As a result, much of what is received by sensory systems fails to be perceived. Despite these limits, certain attributes can help to guide attentional resources and increase the probability that a stimulus will be perceived. One such attribute is the emotional valence of stimuli, particularly when the valence is negative^1–5^. For example, in the attentional blink (AB) task, perception of the second of two consecutive targets (T2) is impaired when the targets are separated by less than approximately 500 ms^6,7^. However, T2 detection greatly improves when it is an emotionally arousing stimulus (e.g. the word “RAPE”) compared to a non-arousing stimulus (e.g. the word “TABLE”)^4,8^. This pattern suggests that emotion may guide attention to stimuli in the environment (emotion guided attention; EGA), which may provide substantial benefits such as aiding in the avoidance of conflict^9^.

Importantly, the extent to which the emotionally arousing qualities of a stimulus guide attention is also modulated by individual differences. For example, adults who report high levels of state and trait anxiety^10^ show higher detection rates when T2 is a fearful face compared to a happy face, while individuals who report low levels of state and trait anxiety show little difference between face types^11^. This pattern echoes a large body of literature that has found relationships between individual differences in anxiety and attention to threat (for a review, see Van Bockstaele *et al*., 2014^12^). By comparison, T2 performance of adults on the autism spectrum does not differ regardless of whether T2 is an emotional word or a neutral word^13,14^ and similar patterns have been found in neurotypical adults with high levels of autistic traits^15^ (though, in child samples, comparative levels of EGA effects have been reported^16^). This pattern echoes a large body of literature that has documented emotional processing difficulties shown by individuals on the autism spectrum^17,18^.

The opposing effects of anxious and autistic traits on EGA are particularly surprising in light of their strong association. Around 29% of autistic individuals are also diagnosed with an anxiety disorder^19,20^. This may be due to shared genetic factors that underlie both disorders^21^ or because difficulties that arise from having autism (e.g. social isolation, discrimination, communication difficulties) contribute to the development of anxiety disorders^20^. Furthermore, measures of autistic traits in neurotypical individuals, such as the Autism Spectrum Quotient (AQ)^22^, are positively correlated (*r* = 0.31–0.65) with measures of trait anxiety^23–25^.

The paradox stemming from the positive correlation between anxiety and autistic traits and their opposing effects on EGA raises important questions as to whether EGA is independently or interactively affected by these two personality traits. For example, when viewing threatening stimuli, how is EGA affected for individuals high in both autistic and anxious traits? Do anxious traits prevail over autistic traits and EGA remains present, or do the emotion processing difficulties associated with autistic traits prevent the typical EGA associated with anxious traits?

Some preliminary investigations have attempted to shed light on this issue by recruiting ASD individuals who vary in terms of anxiety levels and having them complete a dot-probe task to assess EGA (usually to threatening stimuli). The dot-probe paradigm typically involves the brief presentation of two visual stimuli (e.g. faces or words), one neutral and one emotional (e.g., threatening) in tone, prior to the presentation of a probe that appears in the location of one of the stimuli. Participants who respond faster to probes presented in place of threatening relative to neutral stimuli are argued to show greater attention to threat – in other words, greater levels of EGA.

Two studies that used this paradigm reported no differences in EGA between anxious autistic, non-anxious autistic, and non-anxious non-autistic groups of participants, suggesting that anxiety-linked EGA mechanisms fail to activate in the presence of autism^26,27^. However, a third study using a similar task found evidence of greater levels of EGA in an anxious autistic group relative to non-anxious autistic and non-anxious non-autistic groups, who showed equivalent levels of EGA, suggesting that anxiety-linked EGA mechanisms in ASD may be intact after all^28^.

Though the focus of the present paper is on behavioural differences in EGA, further insight can also be gleaned from neuroimaging studies that have looked at the combined effects of autism and anxiety. For example, most neural models suggest that autism is associated with amygdala hypoactivation when viewing stimuli containing emotional information^29–34^ whilst anxiety is associated with amygdala hyperactivation^35–41^. However, a recent functional magnetic resonance imaging study examining amygdala function^42^ revealed that activation while viewing faces was reduced for a subset of autistic children identified with low levels of anxiety relative to a subset of autistic children with high anxiety and also typically developing children not divided on the basis of anxiety levels, who did not differ from one another. This suggests that amygdala hypoactivation usually linked to autism can be masked by comorbid anxiety and that EGA should be relatively intact for autistic individuals with high anxiety but reduced for autistic individuals with low anxiety.

In addition to their heterogenous findings, a significant limitation in many of the previous studies described above is the absence of a full 2 × 2 design comparing autistic/non-autistic and anxious/non-anxious participants^26–28,42^, with most lacking an anxious non-autistic group. Currently, it is unclear whether EGA levels seen in anxious autistic groups is comparable to the EGA levels that may have been expressed by individuals without ASD, but with similar levels of anxiety. Consequently, it is possible that across many of the studies, including the study in which EGA was elevated in the anxious autistic participants^28^, levels of EGA expressed in the anxious autistic groups could be lower relative to levels that would be expressed in an anxious non-autistic group.

In sum, two previous studies used the AB paradigm to investigate EGA, with one reporting elevated EGA as a function of higher levels of anxiety^11^ and the other reporting reduced EGA as a function of higher levels of autistic traits^15^. Anxious and autistic traits have not been investigated together in relation to the EGA under AB conditions. However, research using the dot-probe paradigm suggests a potential interaction in the form of higher levels of autistic traits dampening the influence of higher levels of anxious traits on the EGA. Alternatively, research on amygdala activation suggests an alternative form of interaction in which higher levels of anxious traits may elevate EGA in individuals with high levels of autistic traits who otherwise may show limited EGA. To investigate these possible relationships, we recruited neurotypical individuals to create a 2 × 2 between-subjects design in which participants scored either low or high on the AQ^22^ and either low or high on the trait scale of the Spielberger State-Trait Anxiety Inventory (STAI)^10^. These participants completed an AB task highly similar to that used in a previous study from our laboratory^15^ to examine EGA.

## Method

### Participants

The study was carried out in accordance with procedures approved by the Human Research Ethics Office at the University of Western Australia. Prior to any participation, potential participants were briefed on the nature of the study and all participants gave written informed consent. Participant recruitment involved filling the cells of a 2 × 2 design where participants were grouped according to low or high levels of autistic and anxious traits (i.e. Low AQ / Low STAI, Low AQ / High STAI, High AQ / Low STAI, and High AQ / High STAI).

Effect sizes obtained in previous studies were used as a guide to estimate target sample sizes in the present study. English *et al*.’s (2017)^15^ three-way Lag x Emotion x AQ Group interaction reported a partial eta square of 0.023, which is relatively small by conventional standards^43^. Whilst Fox *et al*. (2005)^11^ did not report the effect size for their three-way Lag x Emotion x STAI Group interaction, we calculated a partial eta square of 0.11 using the MorePower tool^44^. Assuming that the effect size for a four-way Lag x Emotion x AQ Group x STAI group interaction would fall between these two reported values, we used the MorePower tool to determine the minimum sample size required to obtain a partial eta square of 0.05 with a power level of 0.8 for our design and determined that a total sample of at least 120 participants would be necessary^44^.

Initially, 292 undergraduate students from the University of Western Australia completed the AQ and both of the state (STAI-S) and trait (STAI-T) subsections of the STAI in a separate screening procedure. Reliability analyses of the scales was adequate with Cronbach’s alpha reaching 0.83 and 0.93 for the AQ and STAI-T respectively. As AQ scores and STAI-T scores were positively correlated, *r* = 0.49, *p* < 0.001, individuals from the initial pool were selectively recruited to achieve adequate differentiation for a 2 × 2 group design. The mean AQ and STAI-T scores of the initial sample were used as the boundary criteria for recruiting into each of the four cells. Consequently, individuals with AQ scores ≤ 105 were designated Low AQ and the remainder designated High AQ, and individuals with STAI-T scores ≤ 42 were designated Low STAI and the remainder designated High STAI. Mean AQ and STAI-T scores were monitored for the four groups during recruitment to maintain adequate differentiation of the groups while ensuring orthogonality of the two factors (High vs Low AQ and High vs Low STAI). For example, if the mean AQ of the Low AQ / Low STAI group began to fall substantially below the mean AQ of the Low AQ / High STAI group during the recruitment period, efforts were made to target the recruitment of individuals who would ‘re-balance’ the means. Recruitment continued in this way until the initial pool was exhausted.

From the initial pool, 141 participants were recruited into the study. Descriptive statistics for the four groups are provided in Table 1. Confirming adequate differentiation and matching of the groups, an AQ Group (Low vs High) x STAI Group (Low vs High) analysis of variance (ANOVA) conducted on the AQ scores yielded a significant main effect of AQ Group, *F*(1, 137) = 257.63, *p* < 0.001, η_p_^2^ = 0.65, but no main effect of STAI Group or interaction (both *p*s > 0.15, both η_p_^2^s < 0.01). An ANOVA of the same design conducted on the STAI-S and STAI-T scores showed significant main effects of STAI Group (STAI-S: *F*(1, 137) = 261.88, *p* < 0.001, η_p_^2^ = 0.66, STAI-T: *F*(1, 137) = 354.62, *p* < 0.001, η_p_^2^ = 0.72), but no main effect of AQ-Group or interaction (all *p*s > 0.30, all η_p_^2^s < 0.01).

**Table 1:** Descriptive statistics for the four comparison groups.

		Low AQ		High AQ	
		Low STAI	High STAI	Low STAI	High STAI
N		45 (8 male)	30 (11 male)	26 (8 male)	40 (11 male)
Age	Mean (St. Dev.)	20.22 (3.05)	20.20 (2.87)	20.35 (2.13)	20.18 (3.05)
(years)	Min. – Max.	18–29	18–31	17–25	18–30
AQ	Mean (St. Dev.)	94.22 (7.84)	95.27 (8.56)	113.30 (6.86)	115.80 (5.06)
	Min. – Max.	72–105	72–105	106–135	107–125
STAI-S	Mean (St. Dev.)	29.67 (4.72)	47.17 (9.08)	28.42 (5.22)	47.27 (6.71)
	Min. – Max.	20–37	38–74	20–36	38–70
STAI-T	Mean (St. Dev.)	33.76 (5.03)	51.77 (6.54)	35.35 (4.51)	52.10 (5.21)
	Min. – Max.	25–42	43–65	25–42	44–68

### Materials

#### Questionnaires

Autistic traits were assessed using the 50-item self-report Autism Spectrum Quotient (AQ)^22^. The questionnaire uses a four-item forced-choice format, and scoring was done using the 1–4 method introduced by Austin (2005)^45^, with higher scores indicating greater levels of autistic traits. The 1–4 scoring method takes advantage of the range of potentially useful information in each item, thus increasing the variability of total AQ scores compared to the binary scoring system originally proposed for the scale^46^. Anxiety levels were assessed using the state and trait sections (each 20-item self-report) of the State-Trait Anxiety Inventory, which is also scored on a 1–4 scale, with higher scores indicating greater levels of anxiety^10^.

#### Attentional blink task

The attentional blink task used was near identical to the task described in our previous work described earlier^15^. The experimental software was modified to reduce the size of the face stimuli from the previous study to increase task difficulty in response to the relatively high level of accuracy observed in the previous study. Besides this change, the experimental software was not altered in any other way to provide maximum comparability to the previous work.

**Figure 1:**
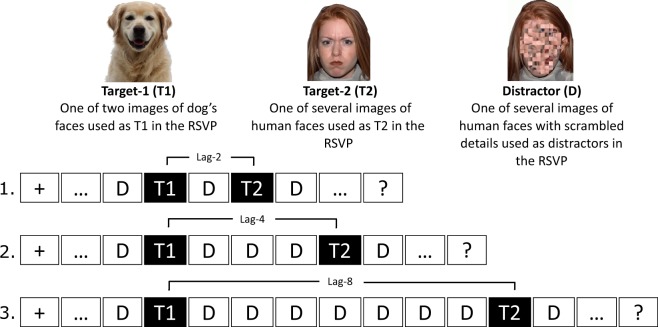
Examples of images used in the RSVP (above) and an illustration of the sequence of images in a (1) lag-2, (2) lag-4 and (3) lag-8 trial (below). Trials began with a fixation cross and ended with prompts about which targets were seen. Catch trials (not illustrated) were identical to test trials except that one or both targets were replaced with a distractor (D) image.

The stimulus sequence on a critical trial, illustrated in Fig. 1, consisted of an initial target (T1; a dog’s face), and a second target (T2; a human face), embedded among distractor stimuli (scrambled faces). Stimuli were presented and responses collecting using Presentation® software (Version 17.0, Neurobehavioral Systems) running on HP EliteOne 800 computers with 23″ displays running at a resolution of 1920 × 1080 and a 60 Hz framerate. Face stimuli were identical to those used in our previous study^15^, consisting of colour headshot photographs selected from the NimStim set^47^ and two colour photographs of dogs’ heads (downloaded from: www.dogbreedinfo.com). All photographs were re-sized to be 1.37° wide × 5.52° high. Three types of stimuli were used in the task: a) the two photographs of dogs’ heads to be used as T1, b) 28 photographs of faces with interior details (e.g. eyes, nose) scrambled and outline preserved to be used as distractors in the rapid serial visual presentation (RSVP), and c) 14 photographs of faces with neutral and angry expressions (seven of each) to be used as T2. These 14 images were half male and half female, and half of the faces had open mouths, while the remaining half had closed mouths. The scrambled faces were generated by Yerys *et al*. (2013)^16^ using a method devised by Conway *et al*. (2008)^48^.

The task consisted of 216 trials divided into three equal blocks, separated by self-paced breaks. Each trial began with the presentation of a fixation cross for 750 ms, followed by a RSVP of 15 images (see Fig. 1). Successive images in the stream were presented at 100 ms intervals (≈17 ms stimulus duration; ≈83 ms blank inter-stimulus interval). Nine different types of trials were presented during the AB task. Three of the types were catch trials with zero or one target, which included: a) 30 T1-only trials, where only the dog’s head was present in the RSVP, b) 30 T2-only trials, where only the unscrambled face was present, and c) 30 trials where neither T1 or T2 were present in the RSVP. The purpose of these trials was to estimate levels of response bias. The remaining six trial types were the test trials that measured the AB and therefore contained both T1 and T2. The six types were formed by crossing three levels of T1-T2 lag with a neutral or angry T2 face.

The first target, if present, was positioned equally often as the fourth, fifth or sixth stimulus in the RSVP. The second target, if present, was positioned as either the second, fourth or eighth stimulus in the RSVP following T1 (lag-2, lag-4 and lag-8 respectively); for T2-only catch trials, T2 was positioned identically following the distractor substituted for T1. Equal numbers of trials were presented at each lag, evenly divided between neutral and angry facial expressions (for test trials, 42 trials per lag; 21 per expression). Presentation order was random with the constraint that identical trial types did not occur more than twice in a row. Each block contained the same number of each trial type. Participants completed ten practice trials before commencing the test trials.

In our earlier study using this paradigm^15^, T2 detection was greater when lag-2 was a face with an angry expression relative to when it was a face with a neutral expression. However, no such difference in performance was present in individuals with greater levels of autistic traits. No difference in T2 detection as a function of emotion was present for either group at lags 4 and 8, possibly due to relatively high detection rates for both emotions at these lags. Thus, while it is expected that lag-2 will be the most sensitive lag for detecting individual differences in emotional guidance in the present study, the other lag conditions were included to replicate the task described in our earlier work^15^ and to allow for detection of any potential effect of anxiety on the emotional guidance of attention at longer lags.

### Procedure

Participants were seated approximately 500 mm in front of the computer monitor. They were instructed to watch closely on each trial for the presentation of a dog (T1) and unscrambled face (T2) in the RSVP stream. Following the RSVP, participants were first prompted to respond if they had observed a dog in the RSVP, and then to indicate whether they had observed an unscrambled face. Participants responded to both queries using ‘Y’ or ‘N’ on the keyboard. Participants were allowed 5000 ms to respond to each of the questions, with no response being recorded as an error. The fixation cross marking the start of the next trial was presented immediately following the second response.

## Results

### Catch trial accuracy

Catch trial accuracy was similar across groups (see Table 2) and every participant achieved a minimum 50% accuracy for each catch trial type. A Catch Trial Type (T1 only vs T2 only vs no target) x AQ Group (Low vs High) x STAI Group (Low vs High) repeated measures analysis of variance (ANOVA) found only a main effect of Catch Trial Type, *F*(2, 274) = 37.29 *p*  < 0.001, η_p_^2^ = 0.21. Follow-up, Bonferroni corrected t-tests revealed that accuracy on T2-only catch trials was significantly lower relative to the other catch trial types (both *p*s  < 0.001, both *d*s > 0.62). The ANOVA revealed two other effects that approached, but did not reach, statistical significance – a main effect of AQ group, *F*(1, 137) = 3.31 *p* = 0.07, η_p_^2^ = 0.24, and a Catch Trial Type x STAI Group interaction, *F*(2, 274) = 2.27 *p* = 0.11, η_p_^2^ = 0.02. The remaining effects did not approach statistical significance, (all *p*s > 0.49, all η_p_^2^s < 0.01), indicating that catch trial performance was largely comparable across participant groups.

**Table 2:** Mean catch trial and Target 1 accuracy (standard deviations in parentheses).

		Low AQ		High AQ	
		Low STAI	High STAI	Low STAI	High STAI
**Mean Accuracy for Catch Trials**					
No Target		94.15% (6.93%)	91.56% (8.15%)	92.18% (7.77%)	90.67% (12.59%)
T1-only		93.26% (7.83%)	94.00% (9.07%)	90.13% (11.45%)	92.83% (9.35%)
T2-only		86.37% (10.75%)	86.78% (11.26%)	82.05% (9.98%)	84.58% (11.31%)
**Mean T1 Accuracy for T1-T2 Trials**					
Lag-2	Neutral	94.39% (7.55%)	94.13% (7.47%)	91.39% (9.62%)	93.69% (11.23%)
	Angry	94.82% (6.57%)	92.86% (8.82%)	92.49% (9.15%)	93.45% (11.70%)
Lag-4	Neutral	94.39% (6.12%)	93.81% (7.83%)	92.13% (7.12%)	95.00% (10.11%)
	Angry	94.29% (6.55%)	95.56% (6.96%)	93.22% (7.40%)	94.64% (7.25%)
Lag-8	Neutral	94.92% (6.13%)	96.03% (5.74%)	94.51% (6.69%)	94.05% (10.88%)
	Angry	94.92% (7.28%)	95.87% (6.47%)	93.77% (7.23%)	94.76% (10.61%)

### T1 accuracy

As can be seen in Table 2, T1 accuracy was uniformly high across conditions. A Lag (lag-2 vs lag-4 vs lag-8) x Emotion (neutral vs angry) x AQ Group (Low vs High) x STAI Group (Low vs High) repeated-measures ANOVA revealed only a significant main effect of Lag, *F*(2, 274) = 6.35, *p* < 0.01, η_p_^2^ = 0.04. Follow-up, Bonferroni corrected t-tests revealed that lag-8 accuracy was significantly greater than Lag-2 accuracy, *t*(137) = 3.12, *p* < 0.01, *d* = 0.27). A further Lag x AQ Group x STAI Group effect approached, but did not reach, statistical significance, *F*(2, 274) = 2.48, *p* = 0.09, η_p_^2^ = 0.02. No other main effects or interactions reached significance (all *p*s > 0.30, all η_p_^2^s < 0.01).

### T2|T1 accuracy

**Figure 2:**
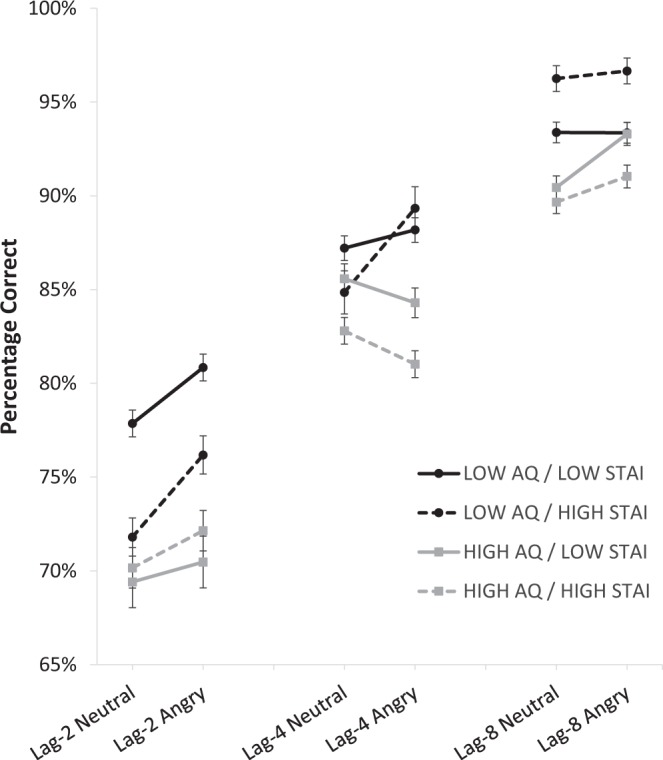
Mean percentage of T2 responses correct given T1 correct, as a function of AQ group (Low and High), STAI group (Low and High), T2 Emotion (neutral and angry) and Lag (2, 4, and 8). Error bars represent within-subjects standard error of the mean. *p < 0.05.

Second-target (T2) accuracy was calculated using only trials on which T1 was correctly identified (i.e. T2|T1) because the source of T2 errors is unclear if a T1 error has also been made^7^. Mean T2|T1 accuracy scores are illustrated in Fig. 2 and were submitted to a Lag x Emotion x AQ Group x STAI Group repeated measures ANOVA. Mauchly’s test indicated that the assumption of sphericity had been violated for effects and interactions involving the within-subjects variable Lag (χ^2^ (2) = 46.09, *p* < 0.001), and so Greenhouse-Geisser estimates of sphericity (ε = 0.77) were applied to the relevant analyses. Assumptions of sphericity were met for effects involving both Lag and Emotion (χ^2^ (2) = 2.85, *p* = 0.24).

This analysis revealed a main effect of Lag, *F*(1.56, 212.82) = 139.42, *p* < 0.001, η_p_^2^ = 0.50, consistent with an attentional blink, and a main effect of Emotion, *F*(1, 137) = 8.75, *p* < 0.01, η_p_^2^ = 0.06, which indicated accuracy was greater for angry faces than neutral faces. There was a main effect of AQ Group, *F*(1, 137) = 4.47, *p* = 0.04, η_p_^2^ = 0.03, such that overall T2|T1 accuracy was higher in the Low AQ group, but there was no main effect of STAI Group, *F*(1, 137) = 0.02, *p* = 0.64, η_p_^2^ < 0.01. Regarding the interactions that were of interest in the present study, the three-way Lag x Emotion x AQ Group effect was significant, *F*(2, 274) = 3.41, *p* = 0.03, η_p_^2^ = 0.02. The interaction corresponds to a relative small effect size^43^ and was comparable to the size of the effect obtained in our previous work^15^. The Lag x AQ Group x STAI Group interaction also approached significance, *F*(1.56, 212.82) = 3.20, *p* = 0.06, η_p_^2^ = 0.01, but was not explored further due to being both uninterpretable and irrelevant to the current study as it lacked an effect of emotion. However, the Lag x Emotion x STAI Group interaction, *F*(1.96, 268.43) = 0.41, *p* = 0.66, η_p_^2^ < 0.01, the Lag x Emotion x AQ Group x STAI Group interaction, F(1.96, 268.43) = 0.27, *p* = 0.76, η_p_^2^ < 0.01, and all other interactions (all *p*s > 0.13, all η_p_^2^ < 0.02) were non-significant. In sum, there was no evidence that STAI Group modulated the relationship between AQ Group and EGA.

We followed up the three-way Lag x Emotion x AQ Group interaction using paired-samples t-tests to test the effect of emotion type on T2|T1 accuracy at each level of Lag and AQ Group. The results of these comparisons are summarized in Table 3, and show that an emotional guidance of attention occurred for Low AQ participants at lag-2 and lag-4, whilst High AQ participants showed only modest emotional guidance of attention at lag-8 (although interpretation of this result is constrained by ceiling levels of performance in all groups).

**Table 3:** Summary of results from paired-sample t-tests conducted to evaluate the simple effect of Emotion for each combination of Lag and AQ Group. Interpretations of Bayes factors are drawn from Jeffreys (1961)^49^.

	t	p	d	BF_01_	BF_10_	Interpretation
**Low AQ**						
Lag-2	3.11	<0.01	0.36	0.10	10.21	Strong for H_1_
Lag-4	2.02	0.047	0.23	1.15	0.87	Anecdotal for H_0_
Lag-8	0.18	0.86	0.02	7.74	0.13	Substantial for H_0_
**High AQ**						
Lag-2	0.93	0.36	0.12	4.90	0.21	Strong for H_0_
Lag-4	−1.46	0.15	0.18	2.70	0.37	Anecdotal for H_0_
Lag-8	2.17	0.03	0.27	0.83	1.20	Anecdotal for H_1_

To provide greater interpretability of these tests, Bayes factors were calculated using *JASP*^50^ and a default Cauchy prior width (*r* = 0.707) that provide the odds ratio for H_0_/H_1_ given the data. Values for *BF*_01_, or the ‘standard’ Bayes factor below 1 are indicative of greater support of H_1_ and values above 1 indicative of support for H_0_. *BF*_10_, or the ‘inverse’ of the Bayes factor, represents the opposite pattern (higher values above 1 indicate support for H_1_, smaller values below 1 indicate support for H_0_). Mathematically, both versions are identical, but we have included both to assist with interpretability (see Jarosz & Wiley, 2014^51^, for an overview of Bayes factors). Jeffreys (1961)^49^ recommended that tests should be associated with *BF* < 0.33 or *BF* > 3 to be statistically meaningful, and his descriptors of *BF*_01_/*BF*_10_ sizes are provided in Table 3 to assist interpretation. Only two comparisons met the criteria for strong evidence in support of either H_0_ or H_1_, with both results occurring at lag-2 when overall T2|T1 performance is lowest and thus the emotional guidance of attention would be expected to be maximal. Strong evidence was found in support of emotional guidance of attention at lag-2 for Low AQ individuals, and for an *absence* of emotional guidance of attention at lag-2 for High AQ individuals. Bayesian t-tests for the remaining comparisons showed relatively weaker levels of evidence in favour of H_0_/H_1_ suggesting other group differences were less reliable.

In summary, these analyses reveal a pattern of results that primarily echo our previous finding of altered EGA in the AB as a function of autistic traits^15^. We did not find evidence that levels of anxious traits affect EGA, whether combined or separated from levels of autistic traits. An additional effect was that accuracy of detection of the face at T2 was lower overall for individuals with high compared to individuals with low levels of autistic traits.

### Correlation and moderation analyses

The analyses reported so far allow for easier comparisons with results from earlier studies by English *et al*. (2017)^15^ and Fox *et al*. (2005)^11^ who used similar statistical analyses. However, the current data also allow for the variables of interest to be treated as continuous variables, allowing for additional analyses to be conducted to support the results of the repeated measures ANOVAs and t-tests outlined earlier. To do this, a single continuous measure of EGA at each level of lag was calculated for each participant by subtracting accuracy scores for neutral trials from accuracy scores for angry trials to obtain a difference score [i.e. angry_lag-2_ – neutral_lag-2_ = EGA_lag-2_], such that larger positive values indicated greater levels of EGA at a given lag.

**Figure 3:**
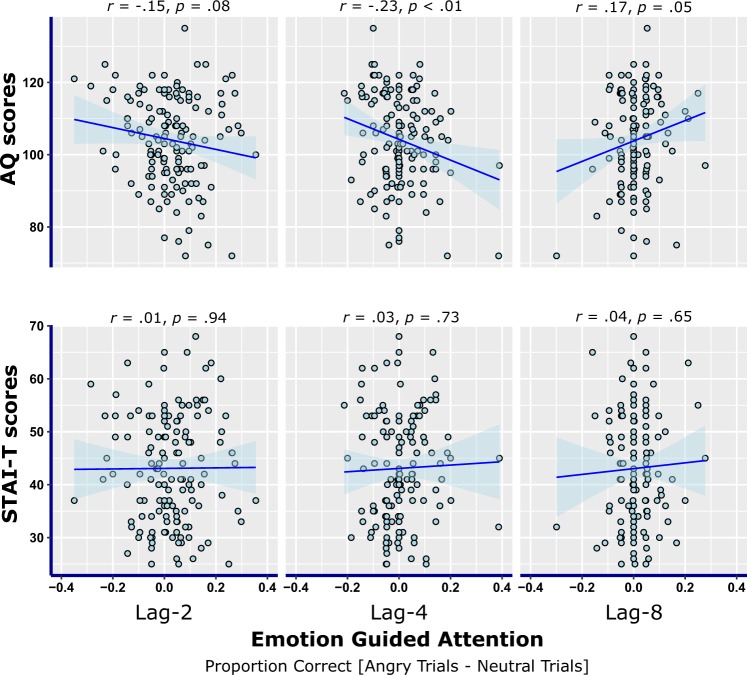
Scatterplots illustrating correlations between autistic traits (top) or anxious traits (bottom) and emotion guided attention difference scores (angry trial accuracy subtract neutral trial accuracy) at each level of lag. Linear functions are fitted to each set of data along with 95% confidence intervals.

Associations between autistic traits, anxious traits and this EGA measure, at each level of lag were first investigated using Pearson’s correlations. Figure 3 outlines the results of the correlation analyses and illustrates them in a series of scatterplots. At each level of lag, variations in AQ scores and EGA are associated (though the effect is marginal for lag-2), while variations in STAI scores and EGA are not. Interestingly, the pattern of data at lag-8 suggests that *higher* levels of autistic traits are associated with superior EGA, although examination of the scatterplots and T2 accuracy scores at lag 8 suggests this is likely due to restriction of range issues arising from ceiling level performance in target accuracy.

By considering continuous data, we were also able to evaluate the possibility that although anxious traits were not found to directly influence EGA at any level of lag, an indirect effect may have been present such that the relationship between autistic traits and EGA was moderated by different levels of trait anxiety. To test this possibility, a series of simple moderation analyses were conducted using the ‘medmod’ add-on for the statistical software package Jamovi^52^ (results were additionally confirmed using Hayes’s PROCESS macro for SPSS^53^). Three analyses were conducted with AQ scores as the dependent variable, STAI-T scores as the moderation variable, and the EGA difference scores calculated as above at each lag as the dependent variable in separate analyses (all variables in the moderation analyses were centred). No moderation effects were found as the AQ x STAI-T interaction was non-significant at each lag (all *p*s > 0.50).

In short, these additional analyses largely support the pattern of data reported by the earlier factorial ANOVAs. We find evidence for a relationship between autistic traits and EGA, though the associations vary in strength and direction at different levels of lag. However, anxious traits do not appear to influence EGA either directly or indirectly.

## Discussion

The purpose of the present study was to better understand how personality traits modulate EGA by resolving an apparent paradox. Autism and anxiety disorders are frequently comorbid with each other^19,20^, and autistic and anxious traits are highly correlated^23,24,54^. However, while individuals with high levels of autistic traits show little-to-no EGA in response to threatening face stimuli in an AB task in contrast to low autistic trait individuals^15^, individuals with high levels of anxiety show greater levels of emotional guidance of attention in the same paradigm relative to low-anxious individuals^11^. To investigate this issue, we selected groups of participants who varied orthogonally in autistic and anxious traits to separately examine the impact of each of these factors on EGA.

The motivation for using this design was that two lines of previous research led us to expect contrasting predictions in relation to either autistic-trait levels moderating the effect of anxious-trait levels on EGA, or alternatively, anxiety-trait levels moderating the effect of autistic-trait levels on EGA. However, neither of these two forms of interaction was observed. Instead, we found a pattern of results that was largely comparable to that reported in English *et al*. (2017)^15^. Low AQ individuals, irrespective of STAI level, detected angry faces more often than neutral faces at lag-2 (the nadir of the AB), and thus demonstrated EGA. In stark contrast, High AQ individuals, again irrespective of STAI level, detected neutral and angry faces with equal accuracy at lag-2, suggesting an absence of EGA. With respect to anxious traits, we were unable to replicate the results outlined by Fox *et al*. (2005)^11^, as comparing the STAI groups produced no differences in EGA.

Based on these results, when it comes to EGA, the effect that high levels of autistic traits has on attentional performance seems to supersede any effect of anxious traits, at least in the context of the AB task and for the extent of separation in anxiety tested here. Of course, it is possible that if clinical and nonclinical levels of anxiety were contrasted (instead of groups differing in trait levels), one might observe differences in EGA as a function of anxiety. However, if we consider autism and anxiety as continua in which traits lie at one end and clinical disorders at the other, an extension of the patterns found in the current data would suggest that autism would have a relatively greater influence on EGA even if clinical levels of anxiety also produce a measurable impact. The present data, combined with knowledge of the high comorbidity of autism and anxiety disorders^19,20^, may have an important bearing on how interventions for improving emotional face processing are conducted for individuals with dual autism and anxiety disorder diagnoses.

With respect to the data suggesting an absence of an effect of anxiety on EGA, one point of difference in methodology between the present study and that of Fox *et al*. (2005)^11^ was the nature of the distractor stimuli. Whereas we used face stimuli that had the details scrambled (see Fig. 1), Fox *et al*. (2005)^11^ used face stimuli with neutral expressions as their distractor images in the RSVP. A second significant point of difference was that participants in the Fox *et al*. (2005)^11^ study had to explicitly identify the emotion of the face presented at T2, whereas participants in our study merely had to indicate whether a face was present or absent. The additional requirement of discriminating emotional faces from neutral faces may have contributed to the influence of anxiety on the EGA in Fox *et al*. (2005)^11^ either because of the explicit requirement to process emotion or because participants needed to engage more perceptual and cognitive resources to complete the task than was required of participants in our study. Finally, sample sizes also differed substantially between the two studies. Whilst we recruited at least 70 participants into each of the Low and High STAI groups, Fox *et al*. (2005)^11^ recruited 14 participants into each of their comparison groups. Thus, it may also be possible that what the previous authors found was a type 1 error, and STAI levels do not predict EGA in an AB paradigm. Adjudicating between these options will require further investigation.

How might the present results reflect the impact of autistic and anxious traits on cortical functioning? The amygdala, cingulate cortex and frontal cortical areas are all implicated in the processing of threatening stimuli presented in an AB paradigm^55,56^. However, it is the amygdala that is likely to be of key relevance to the present work. Imaging studies have shown that amygdala activation increases when individuals view threat-related stimuli, potentially reflecting prioritization of emotionally significant stimuli^57–60^. Moreover, this activation is greater in individuals with high anxiety compared to those with low anxiety when viewing fearful faces^39^. At the same time, a substantial body of evidence suggests that the amygdala suffers developmental failures in autistic individuals (for a review, see Schultz, 2005^61^). Taken together, we speculate that the dominant influence of autistic-traits over anxious traits reflects a reduction in amygdala activity associated with autistic traits, which blunts the usual increase in amygdala activity in response to emotional stimuli seen in anxious individuals. Of course, our behavioural data alone cannot be considered direct evidence of amygdala activation levels and this hypothesis requires further examination using neuroimaging techniques.

In addition to the interaction between AQ Group, Lag and Emotion, the data also showed a significant main effect of AQ Group on T2|T1 accuracy, with High AQ participants less accurate overall in detecting faces compared to Low AQ participants. Importantly, this effect is not problematic with respect to the investigation of the current hypotheses as we were concerned with modulations of EGA as a function of either autistic-trait or anxiety-trait level, rather than with trait group differences in face detection overall. Whilst this effect would imply an association between difficulty in the rapidly processing of faces and elevated levels of autistic traits, including across lags that typically fall outside the AB, we suggest treating this outcome with caution. First, the main effect is compromised by the AQ Group x Lag x Emotion interaction, and second, main effects of participant group were not reported in similar work using emotional word stimuli and adults with/without an ASD^13,14^, work using emotional face stimuli and children with/without ASD^16^, or even our own work using emotional face stimuli and high/low AQ adults^15^.

One possible limitation of the present study is related to our measure of anxiety. To maintain consistency with Fox *et al*.‘s (2005)^11^ methodology, we used the STAI to quantify individuals’ anxiety levels. However, the STAI focuses on general anxiety (e.g. with questions such as “I feel nervous and restless”) rather than social anxiety specifically^10^. It is therefore possible that some participants who reported high STAI scores did not have appreciable levels of social anxiety, and thus, given the social nature of our stimuli, might not be expected to attend differently to threatening and neutral faces. Although this would also be true of Fox *et al*.’s (2005) study^11^, we recommend that future work in this area uses alternative measures to discern between individuals with social and non-social anxious traits.

It is also possible that our pattern of results may have been different if fearful faces were used as the emotional target instead of angry faces. Previous work has found that individuals with high trait anxiety are more sensitive to the gaze direction of fearful faces than faces with other expressions, including anger, whereas low trait anxiety individuals show no differences across emotion type^62^. If using fearful faces were to reveal attentional biases in high-anxiety individuals, then given the current data, we predict that these effects would be restricted to the subset of individuals with Low AQ. This is because this subset has demonstrated the ability to respond to threatening images in an RSVP, whilst High AQ adults in this study, and in our previous study^15^, have not. Such an outcome would still be consistent with our main finding that the presence of autistic traits has a more powerful influence on EGA than anxious traits.

In sum, this study explored the apparently opposing effects of autistic and anxious traits on the EGA in an AB paradigm to better understand the influence of personality traits on attentional capture by emotional stimuli. The main finding was that individual differences in autistic traits influenced whether individuals could use emotional information to guide their attention whereas individual differences in anxiety did not. Notably, the simultaneous exploration of the effects of autistic and anxious traits on attention is relatively uncommon and, given the reported comorbidity between the two factors, further investigation of early attention to emotion may improve our understanding of how individuals diagnosed with autism process face stimuli during social interactions.

## Data Availability

The datasets generated during and/or analysed during the current study are available from the corresponding author on reasonable request. Similarly, the experimental files and stimuli used to conduct the experiment are also available from the corresponding author on reasonable request.
